# Patient Engagement With and Perceptions of the COVIDA Project, a Volunteer-Led Telemonitoring and Teleorientation Service for COVID-19 Community Management: Mixed Methods Study

**DOI:** 10.2196/51237

**Published:** 2024-09-13

**Authors:** Stefan Escobar-Agreda, Javier Silva-Valencia, Percy Soto-Becerra, C Mahony Reategui-Rivera, Kelly De la Cruz-Torralva, Max Chahuara-Rojas, Bruno Hernandez-Iriarte, Daniel Hector Espinoza-Herrera, Carlos Alberto Delgado, Silvana Matassini, Javier Vargas-Herrera, Leonardo Rojas-Mezarina

**Affiliations:** 1 Unidad de Telesalud Facultad de Medicina Universidad Nacional Mayor de San Marcos Lima Peru; 2 Vicerrectorado de Investigación Universidad Continental Lima Peru; 3 Department of Biomedical Informatics School of Medicine University of Utah Salt Lake City, UT United States; 4 Facultad de Medicina Universidad Nacional Mayor de San Marcos Lima Peru; 5 Department of Anthropology University of Southern California Los Angeles, CA United States; 6 Facultad de Salud Pública y Administración Universidad Peruana Cayetano Heredia Lima Peru

**Keywords:** telemonitoring, volunteers, engagement, COVID-19, Peru, telehealth, perceptions

## Abstract

**Background:**

During the pandemic in Peru, the COVIDA (Collaboration Network of Volunteer Brigade Members for the Investigation, Detection, and Primary Management of Community Cases Affected by COVID-19) project proposed an innovative way to provide telemonitoring and teleorientation to COVID-19 patients, led by health care student volunteers. However, it has not been described how this interaction is perceived from the patient’s perspective and which factors increase their engagement with this service.

**Objective:**

The aim of this study is to describe the perceptions of patients about COVIDA and identify factors associated with their engagement with this service.

**Methods:**

A mixed methods study was conducted to evaluate perceptions of patients that participated in the COVIDA project. This telehealth intervention organized by the National University of San Marcos was implemented in Peru from August to December 2020. The service involved daily phone calls by volunteer students to monitor registered COVID-19 patients until the completion of the 14th day of the illness or if a warning sign was identified. The volunteers also provided teleorientation to address the patients’ needs and concerns. Quantitative analysis was performed to describe the characteristics of the patients and to assess the factors related to their engagement with the service, which was defined by the percentage of participants who completed the follow-up according to their individual schedule. Qualitative analysis through semistructured interviews evaluated the patients’ perceptions of the service regarding the aspects of communication, interaction, and technology.

**Results:**

Of the 770 patients enrolled in COVIDA, 422 (55.7%) were female; the median age was 39 (IQR 28-52) years. During the monitoring, 380 patients (49.4%) developed symptoms, and 471 (61.2%) showed warning signs of COVID-19. The overall median for engagement was 93% (IQR 35.7%-100%). Among those patients who did not develop warning signs, engagement was associated with the presence of symptoms (OR 3.04, 95% CI 2.22-4.17), a positive COVID-19 test at the start of follow-up (OR 1.97, 95% CI 1.48-2.61), and the presence of comorbidities (OR 1.83, 95% CI 1.29-2.59). Patients reported that the volunteers provided clear and valuable information and emotional support. Communication via phone calls took place smoothly and without interruptions.

**Conclusions:**

COVIDA represents a well-accepted and well-perceived alternative model for student volunteers to provide telemonitoring, teleorientation, and emotional support to patients with COVID-19 in the context of overwhelmed demand for health care services. The deployment of this kind of intervention should be prioritized among patients with symptoms and comorbidities, as they show more engagement with these services.

## Introduction

The COVID-19 pandemic has posed a significant public health challenge due to its widespread impact and COVID-19’s capacity to lead to severe cases and fatalities in the population [1]. While timely medical care has been shown to prevent such outcomes [2], patients encounter challenges in seeking care due to difficulties in self-monitoring caused by a lack of knowledge of their symptoms and the possibility of developing severe conditions even without symptoms [3].

In this context, telemonitoring has emerged as an essential alternative to facilitate follow-up among patients with COVID-19 using information and communication technology (ICT) [4]. In Peru, one of the countries most affected by this disease worldwide [5], the government implemented some institutional initiatives to promote remote monitoring of these patients [6]. However, despite the high demand for services, the implementation of these interventions was limited by the lack of availability of health workers, for reasons including their loss during the pandemic [7].

Considering the willingness of health sciences students to participate in health interventions using ICT [8] and provide assistance in the context of emergencies [9], the Telehealth Unit of the National University of San Marcos (UNMSM) and the Peruvian National Institute of Health (INS-Peru) collaborated to design and implement the COVIDA (Collaboration Network of Volunteer Brigade Members for the Investigation, Detection, and Primary Management of Community Cases Affected by COVID-19) project. This intervention was a telemonitoring and teleorientation service carried out by university student volunteers and supervised by a team of health care professionals [10] that aimed at the prompt identification of severe signs related to COVID-19.

Volunteer-based services for the remote monitoring of patients during the COVID-19 pandemic have been found helpful in the international context [11,12]. However, there is little evidence on this kind of intervention in resource-limited settings [13] or on patients’ perspectives and level of engagement with aspects of these services [14] that are crucial to develop sustainable and patient-centered telehealth solutions that met their needs and preferences [15]. This study aims to describe the level of engagement and patients’ perceptions of their participation in a telemonitoring and teleorientation service (the COVIDA project) in Peru.

## Methods

### Study Design

A mixed methods study with an explanatory sequential design was conducted, as is considered best practice in evaluating telehealth interventions [16]. First, a quantitative analysis was performed to gather information on patient characteristics and their level of engagement with the service. Then, a qualitative analysis was conducted to amplify the patient and volunteer experiences, providing deeper insights from the perspectives of both patients and health care providers.

### The COVIDA Project

#### Design and Services

**Figure 1:**
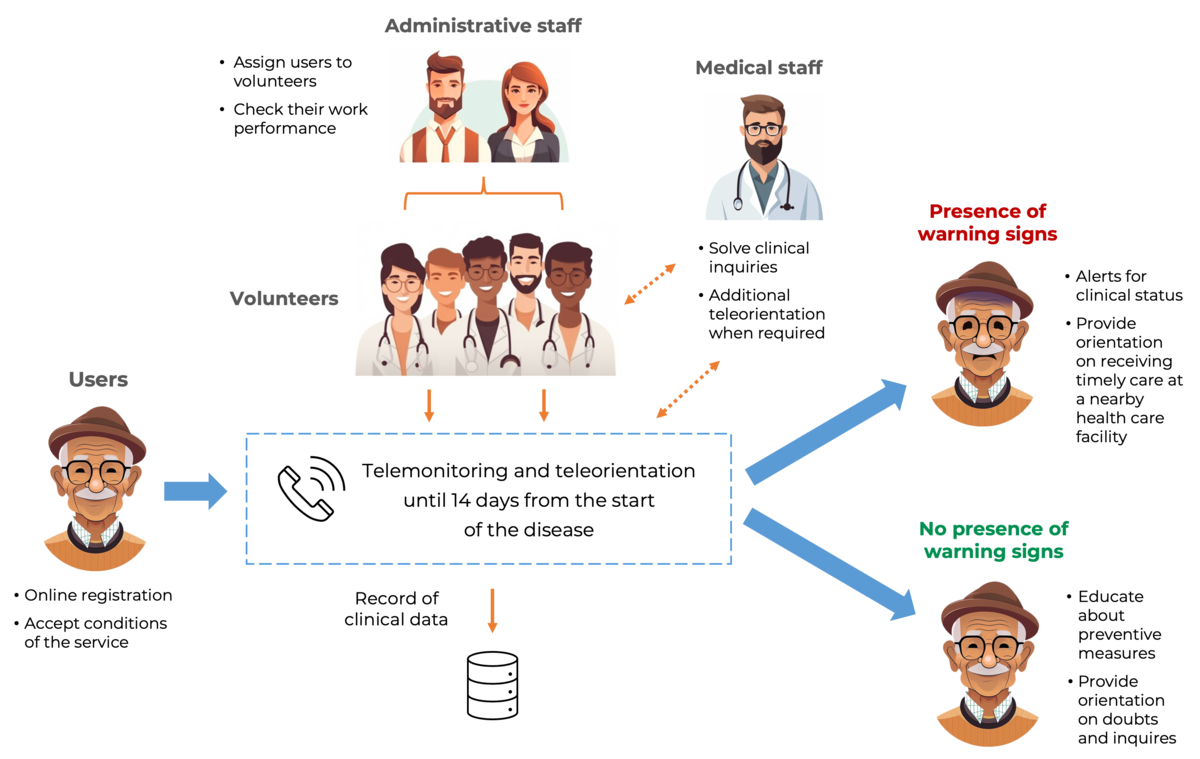
Design of COVIDA (Collaboration Network of Volunteer Brigade Members for the Investigation, Detection, and Primary Management of Community Cases Affected by COVID-19) for the telemonitoring and teleorientation of patients with COVID-19 in Peru. Warning signs included shortness of breath, chest pain, persistent fever, cyanosis, or having an oxygen saturation less than 93% (among patients with a pulse oximeter).

The COVIDA project consisted of a telemonitoring and teleorientation service provided in Peru between September to December 2020 after the end of the first wave of infections of COVID-19 in this country. The telemonitoring component involved daily phone calls to identify those with suspicions of developing severe COVID-19 symptoms based on warning signs, such as shortness of breath, chest pain, persistent fever, cyanosis, or low oxygen saturation levels (less than 93% when the patient had a pulse oximeter), following the World Health Organization guidelines [17]. Sociodemographic and clinical information from participants was registered in online forms using a free software application called KoboCollect (Kobo, Inc). Additionally, volunteers provided teleorientation to educate patients on adopting preventive behaviors, avoiding unsupervised medication, resolving their doubts and misconceptions about COVID-19, and guiding them to seek timely medical care when in the presence of warning signs. This teleorientation was provided via phone calls that were placed during the day according to the patient’s availability and were expected to continue until the end of 14 days from the start of their illness, which was determined by the date of symptom onset, a recent positive test result, or the development of a warning sign (Figure 1).

#### Patients, Volunteers, and Support Team

The COVIDA project was designed for adult patients (aged ≥18 years) in Peru who had tested positive for COVID-19, had related symptoms of the disease (Multimedia Appendix 1, Table S1), or had close contact with a positive case. The volunteers that provided the service were undergraduate or recent-graduate health students who received training from INS-Peru on essential aspects of COVID-19, communication skills in emergencies, and informatics applications for calls and virtual surveys. Patients and volunteers were recruited through social media, online newspapers, and the websites of UNMSM and INS-Peru [10,18].

Additionally, this intervention included an administrative support team in charge of gathering information on registered patients, assigning them to volunteers according to their availability, and checking their work performance, as well as a medical staff that oversaw inquiries made by the volunteers and provided additional teleorientation to patients with complex cases or when a patient requested (Figure 1).

### Variables and Data Collection

For this study, we collected quantitative data from the telemonitoring registers of the COVIDA project provided by UNMSM. We used these data to describe the characteristics of the patients who participated in this service and their level of engagement. The characteristics evaluated included age, sex, region of origin, health insurance, presence of comorbidities, number of days of telemonitoring, development of symptoms, and development of warning signs. We defined the level of patient engagement as the percentage of days on which the patient was effectively telemonitored according to what was planned for each case (see the Design and Services section).

We also conducted semistructured interviews with patients to explore their experiences and perceptions about their participation in the COVIDA project. The interviews were conducted via phone calls after the conclusion of the service and lasted approximately 10 to 15 minutes. We focused on evaluating 3 main aspects of their experience: communication, interaction, and technological aspects.

### Analysis

#### Quantitative Component

We performed descriptive analyses of the sociodemographic and clinical characteristics of the patients who participated in the COVIDA project. Numerical data were summarized as means and SDs. For categorical data, we used frequencies and proportions. Bivariate analyses were performed to compare the characteristics of patients who developed warning signs with those who did not. We used the *χ*^2^ test or Fisher exact test (if the data did not meet the Cochrane criteria) to compare categorical data and a 2-tailed Student *t* test to compare numerical data.

Multivariable analysis was performed to evaluate patient characteristics that were related to higher engagement with the service among patients that did not develop warning signs. Considering that engagement was an outcome that was bound between 0 and 1, a fractional regression model was applied to calculate odds ratio (ORs) with the 95% CI using crude, fully adjusted, and parsimonious models. Detailed information about these analyses is provided in Multimedia Appendix 1. All analyses were conducted using Stata IC (version 17; StataCorp).

#### Qualitative Component

Five patients who agreed to interviews were selected. Semistructured interviews were conducted, recorded, transcribed, and compiled into a matrix for further thematic analysis [19].

### Ethical Considerations

This study was approved by the Social Security (EsSalud) Institutional Review Board for COVID (ethics certificate 96; June 2020). This study was carried out respecting the Helsinki and Taipei declarations on research principles. The quantitative analysis involved secondary data from the COVIDA project provided by the UNMSM. These data were fully deidentified, ensuring that no personal information could be traced back to any individual and allowing all analyses to be conducted anonymously. For the qualitative component, participants were enrolled after completing an informed consent form, with their information managed confidentially and accessed only by the researchers involved in the study. Participants were informed of the study’s purpose, and interviews were scheduled at a time convenient for them to avoid disrupting their daily or work activities. As a result, no financial compensation was provided.

## Results

### Quantitative Results

Of the 1794 individuals who initially registered for COVIDA, 670 (37.3%) had a documented history of telemonitoring. In addition, telemonitoring records were identified for 108 patients who where not initially registered in the system. Finally, 8 of them were excluded due to being younger than 18 years, resulting in total cohort of 770 patients included in the analysis. The patients had a median age of 39 (IQR 28-52) years and were monitored for a median of 2 (IQR 1-4) days. A total of 422 (55.7%) were female, 474 (64.2%) were from Lima-Callao (the capital city), 523 (78.5%) reported having some form of health insurance, and 171 (22.2%) reported having at least 1 comorbidity (detailed clinical information is shown in Multimedia Appendix 1, Tables S1 and S2). The characteristics of those that developed and did not develop warning signs during telemonitoring were similar, except for the presence of comorbidities, which was more common in those that developed warning signs (*P*<.001; Table 1).

**Table 1:** Characteristics of participants in the COVIDA (Collaboration Network of Volunteer Brigade Members for the Investigation, Detection, and Primary Management of Community Cases Affected by COVID-19) project (Peru, 2020) overall and according to the development of warning signs during telemonitoring.

		All patients	With alarm signs	Without alarm signs	*P* value	
Total, n (%)		770 (100)	241 (31.3)	529 (68.7)	.20^a^	
Age (years), median (IQR)		39.0 (28-52)	41.0 (29-51)	38.0 (28-53)	<.001^a^	
Telemonitoring time (days), median (IQR)		2.0 (1-4)	1.0 (1-3)	2.0 (1-4)		
**Age categories (years), n (%)**						.48^b^
	18-59	650 (86.1)	442 (85.5)	208 (87.4)		
	≥60	105 (13.9)	75 (14.5)	30 (12.6)		
**Sex**						.25^b^
	Male	335 (44.3)	98 (41.2)	237 (45.7)		
	Female	422 (55.7)	140 (58.8)	282 (54.3)		
**Macroregion of origin, n (%)**						.56^c^
	Lima-Callao	474 (64.2)	138 (60.3)	335 (65.8)		
	Center	66 (8.9)	22 (9.6)	43 (8.4)		
	North	126 (17.1)	47 (20.5)	81 (15.9)		
	South	61 (8.3)	19 (8.3)	42 (8.3)		
	West	11 (1.5)	3 (1.3)	8 (1.6)		
**Health insurance, n (%)**						.91^b^
	No	143 (21.5)	44 (21.1)	98 (21.4)		
	Yes	523 (78.5)	165 (78.9)	359 (78.6)		
**Presence of comorbidities, n (%)**						<.001^b^
	No	599 (77.8)	165 (68.5)	434 (82)		
	Yes	171 (22.2)	76 (31.5)	95 (18)		

^a^Mann-Whitney U test.

^b^*χ*^2^ test.

^c^Fisher exact test.

Patients in the COVIDA project had a median patient engagement of 93% (IQR 36%-100%). Among those who did not develop warning signs, the factors related to a higher engagement with the service were the presence of comorbidities (OR 1.79, 95% CI 1.27-2.53), having a recent positive test for COVID-19 at registration (OR 1.99, 95% CI 1.51-2.62), or having COVID-19–related symptoms at registration (OR 2.96, 95% CI 2.18-4.01) (Table 2).

**Table 2:** Relationship of patient engagement (%) and sociodemographic and clinical characteristics among patients who did not develop warning signs during the COVIDA (Collaboration Network of Volunteer Brigade Members for the Investigation, Detection, and Primary Management of Community Cases Affected by COVID-19) project.

Characteristics			Patient engagement (%)				
			Crude OR (95% CI)		Model 1^a^: fully adjusted OR (95% CI)		Model 2: parsimonious OR (95% CI)
**Age categories (years)**							
	18-59	Reference		Reference		—^b^	
	≥60	0.88 (0.59-1.31)		0.92 (0.65-1.33)		—	
**Sex**							
	Male	Reference		Reference		—	
	Female	1.09 (0.85-1.40)		1.06 (0.80-1.40)		—	
**Region**							
	Lima-Callao	Reference		Reference		—	
	Center	0.54 (0.35-0.84)		0.69 (0.40-1.16)		—	
	North	0.92 (0.66-1.30)		1.15 (0.79-1.66)		—	
	South	0.68 (0.43-1.06)		1.33 (0.80-2.22)		—	
	West	0.61 (0.20-1.84)		0.99 (0.20-4.78)		—	
**Health insurance**							
	No	Reference		Reference		—	
	Yes	1.26 (0.92-1.74)		1.13 (0.81-1.56)		—	
**Have comorbidities**							
	No	Reference		Reference		—	
	Yes	2.22 (1.59-3.10)		1.83 (1.29-2.59)		1.79 (1.27-2.53)	
**Have positive test at start**							
	No	Reference		Reference		—	
	Yes	2.69 (2.09-3.46)		1.97 (1.48-2.61)		1.99 (1.51-2.62)	
**Have symptoms at start**							
	No	Reference		Reference		—	
	Yes	3.13 (2.29-4.27)		3.04 (2.22-4.17)		2.96 (2.18-4.01)	

^a^Model 1 included covariables based on theoretical background.

^b^Not applicable.

### Qualitative Results

The following insights stem from a thematic analysis conducted to explore patients’ perspectives on various facets of volunteer services. Three main themes emerged, shedding light on the services’ communicational, interactive, and technological aspects.

#### Communicational Aspects: Clarity and Compassion

On the communicational aspects, patients reported that volunteers provided clear information using simple language. In addition, patients highlighted their willingness, kindness, and patience to provide explanations. In some cases, volunteers became emotional companions, offering comfort during anxiety and sadness related to the illness.

The volunteers attend you with patience and kindness, although I asked the same questions, they did not hesitate to explain you again and nice so that you understand...when I complained that I was worried they were also kind.

Patient 3

The lady who attended me, apart from worrying about my physical health, listened to me, I think she has been an important support, they were difficult times, and that someone listens to you helps you a lot, so I am grateful.

Patient 2

#### Interactive Aspects: Challenges and Misconceptions

The interactive aspects of the service posed some challenges, as some patients needed clarification on whether the volunteer labor was supervised or if the volunteers were doctors. We supposed that these initial misconceptions were barriers to volunteer work.

I knew they were medical students from San Marcos, but I didn’t understand how it worked, I think they are supervised by doctors, but it has been a good service.

Patient 1

In addition, some patients reported annoyance toward the frequent calls, including those made during nighttime hours, despite having initially agreed to this arrangement. This was more common in those who were asymptomatic or had milder symptoms.

I know they wanted to help us, but sometimes they called you late and asked you the same thing again, that generated a bit of annoyance to me.

Patient 4

I think that if you were not so serious, they should not call you so much, just every couple of days, because we are also busy or else, they should notify us in advance at what time they would call us.

Patient 5

#### Technological Aspects

Finally, regarding the technological aspects, patients did not report any significant issues and stated they communicated smoothly with volunteers without interruptions.

I have been able to communicate fluently with the volunteers, there were no inconveniences in the phone calls.

Patient 2

## Discussion

The COVIDA project, a volunteer-led telemonitoring and teleorientation service for COVID-19 patients, has been met with a positive reception and a high level of patient engagement. A contributing factor to this success appears to be the effective communication between patients and volunteers, facilitated by the simplicity of the technology used, which relied on phone calls. This approach enabled the delivery of consistent monitoring quality to patients regardless of their access to pulse oximeters, as these demand some considerations for their proper use [20] and require internet connectivity, which can be notably limited in countries such as Peru [21], and necessitate proficient use of digital devices [4], a skill that may be lacking due to low digital literacy levels in our population, as observed in older adults [22]. Notably, no technical challenges were reported by any patient involved in our study during the monitoring period.

Another factor that might have contributed to the strong engagement with the COVIDA project is the integration of teleorientation and support alongside telemonitoring. Interviewed patients mentioned that this service offered clear and useful information about COVID-19 and facilitated a space where they felt listened to and received emotional support to encompass challenging times. These aspects could potentially contribute to diminishing the psychological distress experienced by COVID-19 patients, which is often associated with their uncertainty about the illness [23,24]. This finding is consistent with the study of Chakeri et al [25] in 2020, which found that interventions designed to provide information and maintain communication with COVID-19 patients can significantly reduce their levels of stress and anxiety.

None of the sociodemographic characteristics of the patients in our study were related to their level of engagement with the service, which contrasts with other international studies that observed higher levels of engagement among older adults compared to younger adults. In our country this relationship may be attenuated by the low level of digital literacy of older adults, as discussed previously [22]. On the other hand, patients with clinical characteristics such as the presence of symptoms, comorbidities, and a positive confirmatory test at the start of telemonitoring showed an increased level of engagement. This could be due to the presence of more warning signs in patients with comorbidities. Additionally, a sense of intrusiveness was reported in interviews with asymptomatic patients regarding the daily monitoring schedule, which could increase their probability of dropping out of the service. These findings suggest the need for offering varying schedules for telemonitoring based on patient preferences, focusing more on patients with risk factors or those who are symptomatic. Moreover, complementing remote monitoring with medical devices, when available, could improve engagement among asymptomatic patients or those who may feel annoyed with frequent calls [26].

The involvement of students as volunteers plays a pivotal role in the sustainability of the COVIDA project. Previous studies have shown that students are a valuable asset in community health initiatives due to their willingness to engage in such endeavors [27,28]. During the first year of the COVID-19 pandemic in Peru, the availability of students increased as numerous educational activities in hospitals were suspended due to the high risk of contracting the disease [29]. While other telemonitoring programs have depended exclusively on the participation of health professionals [30], this initiative has shown that, with adequate training and supervision, these activities could be performed by students in health career programs. This approach is particularly significant in contexts where there is a limited availability of human resources for health, such as Peru, which experienced significant disruptions and constraints in the health care workforce during the COVID-19 pandemic [7].

The participation of administrative and clinical staff was another important aspect of the sustainability of the COVIDA project. As the telemonitoring and teleorientation activities were carried out voluntarily by students, the availability of some of them could have been modified or suspended during the intervention, affecting the continuity and quality of the monitoring provided. For this reason, the administrative staff were responsible for assigning patients to the volunteers, confirming their availability daily, and reassigning work to another volunteer if necessary. Additionally, the clinical staff clarified any doubts the volunteers had about using the online tools available in the intervention or aspects of COVID-19. They also provided additional teleorientation to patients with complex cases or to others requiring it. This support was essential to ensure the smooth running of the project and the provision of high-quality monitoring to patients.

This study has some limitations that need to be highlighted. First, there was a high rate of participants with no telemonitoring information among those initially registered. According to the data provided for analysis, some telemonitored patients had no initial registration. This could be due to misregistration of the identification code of patients. In COVIDA, patient identification codes were the number of their national identification document registered manually on each telemonitoring contact. Using web-based forms specialized for the follow-up of patients could have helped prevent the loss of information in telemonitoring interventions caused by misregistration of identifiers. Despite these limitations, our study demonstrated that the COVIDA project was an affordable and well-accepted initiative in identifying and guiding people suspected of developing severe COVID-19 in Peru. The project was also a sustainable intervention in its context, with an overwhelming demand for attention and limited economic and human resources.

The COVIDA project has presented a promising model for providing telemonitoring and teleorientation services for COVID-19 that has been well received and is sustainable. Given its characteristics, it could be adapted to monitoring other diseases where a severe or fatal outcome should be prevented in the short term, such as diabetes or hypertension. However, further research is needed to evaluate the clinical effectiveness of such interventions in preventing severe outcomes like hospitalization, intensive care unit admission, and death. Additionally, it is crucial to address scalability issues in future studies to ensure that similar interventions can be implemented on a larger scale and in other settings. Overall, the COVIDA project has demonstrated the potential of telemonitoring and teleorientation in resource-constrained settings and highlights the need for continued research and innovation in this area.

## Appendix

Multimedia Appendix 1 Supplementary information and Tables S1-S4.

## Abbreviations

COVIDA: Collaboration Network of Volunteer Brigade Members for the Investigation, Detection, and Primary Management of Community Cases Affected by COVID-19
ICT: information and communication technology
INS-Peru: Peruvian National Institute of Health
UNMSM: National University of San Marcos
